# Guava Leaf Extract as a Sustainable Preservative Alternative in Semi-Solid Pharmaceuticals: Efficacy and Stability Assessment

**DOI:** 10.3390/antibiotics14121176

**Published:** 2025-11-21

**Authors:** Hamada Imtara, Mohammad Atiya, Michel Hanania, Jehad Abbadi, Samer Mudalal, Fuad Al-Rimawi

**Affiliations:** 1 Department of Biology and Biochemistry, Faculty of Medicine, Arab American University, Ramallah P600, Palestine; hamada.imtara@aaup.edu; 2 Chemistry Department, Faculty of Science and Technology, Al-Quds University, Jerusalem P.O. Box 20002, Palestine; mohammad.atiya@students.alquds.edu; 3 Chemistry Department, Faculty of Science and Technology, Bethlehem University, Bethlehem 00970, Palestine; mhanania@bethlehem.edu; 4 Biology Department, Faculty of Science and Technology, Al-Quds University, Jerusalem P.O. Box 20002, Palestine; jabbadi@staff.alquds.edu; 5 Nutrition and Food Technology Program, Department of Agricultural Engineering, Faculty of Veterinary Medicine and Agricultural Engineering, An-Najah National University, Nablus P.O. Box 7, Palestine

**Keywords:** *Psidium guajava* (L.), pharmaceutical formulations, clotrimazole cream and ibuprofen gel

## Abstract

**Background**: Natural alternatives are becoming more popular as a result of health risks associated with synthetic preservatives in pharmaceuticals. Because of its antibacterial properties, *Psidium guajava* (L.) leaf extract is a practical choice for sustainable preservation. **Objectives**: The aim of this research is to evaluate the efficacy of *Psidium guajava* (L.) leaf extract as a 10% (*w*/*w*) natural preservative in five different base formulations: creams (clotrimazole cream, permethrin cream, and gentamicin cream) and gels (indomethacin emulgel and ibuprofen gel). **Methods**: Over the course of 28 days, antimicrobial activity against *Pseudomonas aeruginosa*, *Staphylococcus aureus*, *Escherichia coli*, *Aspergillus brasiliensis*, and *Candida albicans* was evaluated. In accordance with USP/BP guidelines, three months of accelerated conditions (40 ± 2 °C/75% ± 5% RH) were used to assess chemical, physical, and microbiological stability. The stability of the active component was examined using HPLC. **Results**: Our findings showed that the extract completely inhibited the growth of tested bacterial species in clotrimazole cream, permethrin cream, gentamicin cream, and indomethacin emulgel, demonstrating significant antibacterial activity across all formulations. However, the antifungal activity was restricted. The preservation effectiveness criterion (bacterial/yeast counts < 10 CFU, mold < 20 CFU) was only fulfilled by ibuprofen gel and clotrimazole cream. Although there were color changes, stability tests verified that the active components such as ibuprofen (reduced from 97.5% to 92%) and clotrimazole (reduced from 99.9% to 95%) decomposed minimally and had acceptable physical characteristics. Particularly against fungus, the extract was surpassed by chemical preservatives (methyl/propyl paraben). **Conclusions**: Certain semi-solid pharmaceuticals can benefit from the stability and antimicrobial protection provided by guava leaf extract, a natural preservative. The weak antifungal activity of the guava leaf extract emphasizes the necessity for specific improvements in the formulation.

## 1. Introduction

Although topical treatments, particularly those in multi-use packaging, are prone to contamination with repeated application, the skin serves as a natural barrier against microbial invasion [1,2,3,4,5,6]. Despite this, skin is prone to various disorders, including inflammation and skin infections caused by different types of bacteria, fungi, or parasites [7,8]. These disorders are often treated with topical medications such as creams and gels [9], which are pharmaceutical formulations composed of several ingredients, such as the active molecules and other substances called excipients [10]. To preserve the composition of active ingredients in pharmaceutical formulations, it is necessary to use preservatives [11] such as parabens [12]. However, artificial preservatives can disrupt hormonal function, in particular the female hormone estrogen [13,14]. The side effects of these preservatives have led to growing interest in using natural preservatives derived from plant extracts as an alternative, as they are less harmful than chemical substances [15].

A number of studies have already been carried out on the use of plant derivatives or extracts as preservatives, with certain plants having demonstrated antimicrobial and antifungal properties [16,17]. It was found that *Psidium guajava* (L.) had several pharmacological activities [18], particularly antimicrobial activity [19].

The medicinal plant Psidium guajava, or guava, has been extensively researched and prized for its diverse phytochemical composition. Various bioactive components, including flavonoids (including quercetin, guaijaverin, and kaempferol), tannins, saponins, triterpenoids, and essential oils, have been found in guava leaf and fruit extracts across numerous investigations [16,17]. The plant’s antibacterial, antioxidant, and anti-inflammatory properties are thought to be mostly attributed to flavonoids and tannins, which are especially prevalent in ethanolic and methanolic extracts [18]. A similar antimicrobial profile has been reported across several investigations; however, the presence of these chemicals may vary based on the extraction process, plant part employed, and geographical origin [19].

Flavonoids and tannins, in particular, are the phytochemicals that work together to give Psidium guajava extract its antibacterial properties. Quercetin is one flavonoid that can interfere with DNA replication by rupturing bacterial membranes and inhibiting enzymes like DNA gyrase. By using metal ion chelation, tannins can deprive microorganisms of vital nutrients and inactivate bacterial proteins and enzymes. All of these behaviors affect the development and survival of bacteria [18,20,21].

Semi-solid formulations should be free from microbial contamination during production, storage, and use; preservative effectiveness testing is crucial, especially for formulations that are given in numerous doses. Because active pharmaceutical ingredients (APIs) have frequently selective or narrow-spectrum activity, additional preservatives are needed even in formulations that contain APIs with antimicrobial activities. On the other hand, preservatives are made to prevent the activity of variety of bacteria, fungi, and yeasts, guaranteeing the product’s safety and effectiveness during its whole shelf life [10,11,15].

Our study is based primarily on the development of several formulations for cutaneous treatment. The formulations were clotrimazole cream for the treatment of vulvar infections caused by vulvovaginal fungi such as Candida, a permethrin cream for the antiparasitic treatment of external infections, and a gentamicin cream as an antibiotic against *staphylococci*. The other two formulations were an indomethacin emulgel and an ibuprofen gel as anti-inflammatory agents. The antibacterial (*S. aureus*: ATCC 6538, *E. coli*: ATCC 8739, and *P. aeruginosa*: ATCC 9027) and antifungal activity (*C. albicans*: ATCC 10231 and *A. brasiliensis*: ATCC 16404) of *Psidium guajava* (L.) extract (10%) were tested in all five formulations. In our study, high-performance liquid chromatography (HPLC) was used to detect and determine the concentration of active ingredients present in the formulations. Finally, the stability of these pharmaceutical products, in the form of creams and gels, was assessed for three months under accelerated conditions (40°/75% RH).

## 2. Results

### 2.1. Chemical Characterization of Psidium guajava (L.) Extract

**Figure 1:**
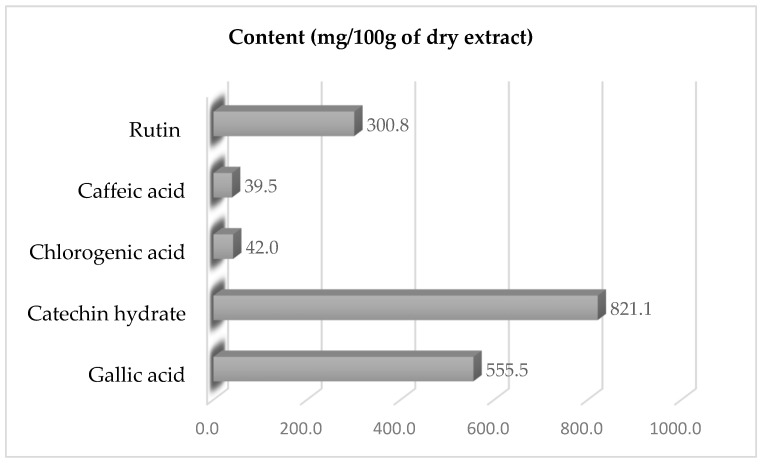
Concentrations of specific phenolic substances in *Psidium guajava* (L.) extract.

The extract contained several essential compounds, of which five are in the majority: gallic acid (555.49 mg/100 g), hydrated catechin (821.11 mg/100 g), caffeic acid (39.54 mg/100 g), hydrated rutin (300.84 mg/100 g), and chlorogenic acid (42.04 mg/100 g) (Figure 1).

### 2.2. Antimicrobial Efficacy Test of 10% P. guajava (L.) Leaf Extract Powder in Five Semi-Solid Pharmaceutical Preparations

The results presented in Table 1, Table 2, Table 3, Table 4 and Table 5 show the pharmaceutical products formulated with a biological preservative based on *Psidium guajava* (L.) leaf extract at a concentration of 10%. The usage of *Psidium guajava* (L.) leaf extract in clotrimazole cream was able to cause complete inhibition of *S. aureus* and *P. aeruginosa* during the tested storage period. There was about a 4.5 log-cycle reduction in the initial load of *E. coli*. *Psidium guajava* (L.) leaf extract at a concentration of 10% showed a strong inhibitory effect against the tested fungi (Table 1). There was a complete inhibitory effect for *Candida albicans* and *A. brasiliensis*.

Inclusion of *Psidium guajava* (L.) leaf extract at a concentration of 10% in permethrin cream showed complete inhibition of all tested bacterial strains (Table 2). On the other hand, the same extract in the same product showed very a mild effect against tested fungal strains.

Similarly to permethrin cream, employing *Psidium guajava* (L.) leaf extract at a concentration of 10% in gentamycin cream led to complete inhibition of all tested bacterial strains (*S. aureus*, *P. aeruginosa*, and *E. coli*). On the contrary, *Psidium guajava* (L.) leaf extract had no antifungal properties in the tested products (Table 3).

Similarly to the previous products and almost in a similar pattern, using *Psidium guajava* (L.) leaf extract (10%) in indomethacin emulgel completely inactivated all tested bacterial strains (*S. aureus*, *P. aeruginosa*, and *E. coli*) at the end of the storage period (28 days) (Table 4). *Candida albicans* and *A. brasiliensis* were not affected by the extract.

A preservative efficacy test for ibuprofen gel containing 10% *P. guajava* leaf extract exhibited a strong inhibitory effect against the growth of *S. aureus* and *E. coli* (complete bacterial destruction at the end of storage time) (Table 5). The count of *P. aeruginosa* was reduced by 3.2 logs. The activity of *Candida albicans* and *A. brasiliensis* was not affected by the addition of 10% *P. guajava* leaf extract.

### 2.3. Pharmaceutical Preparations with Natural Preservative (10% P. guajava Leaf Extract) in Comparison to Pharmaceutical Preparations with Chemical Preservatives (Methyl and Propyl Paraben)

The results presented in Table 6 show the efficacy of the natural preservative (*P. guajava*) compared to chemical preservatives (methyl and propyl paraben) in clotrimazole cream. Overall, our results show that the effectiveness of the natural preservative was comparable to the chemical preservatives for all tested microorganisms except *E. coli*. In this context, *E. coli* was reduced by about 3.5 logs using a natural preservative, while by using a chemical preservative, there was complete inhibition of the growth of microorganisms. Moreover, chemical preservatives were faster than the natural preservative in the destruction of tested microorganisms (complete inhibition: first week vs. second week).

The results related to the efficacy of natural preservatives (*P. guajava*) compared to chemical preservatives (methyl and propyl paraben) in ibuprofen gel are shown in Table 7. It was found that the antibacterial activity of natural preservatives was similar to the chemical preservatives for all tested bacterial strains. Natural preservatives exhibited very weak antifungal activity, while chemical preservatives showed very strong antifungal activity. *Candida albicans* was completely inhibited by the chemical preservatives, while the count of *Candida albicans* was reduced by 2.25 logs using a natural preservative. The activity of *A. brasiliensis* was not affected by using a natural preservative.

### 2.4. Stability Test of Pharmaceutical Preparations

#### 2.4.1. Characterization of Ibuprofen Gel, Clotrimazole Cream, and Permethrin Cream by HPLC

Ibuprofen gel and clotrimazole cream were chosen as high-risk cream and gel examples for chemical stability benchmarking. Given matched excipient systems and preservation mechanisms, ICH Q1D bracketing principles facilitate extrapolation to analogous formulations (gentamicin/permethrin creams and indomethacin emulgel).

**Figure 2:**
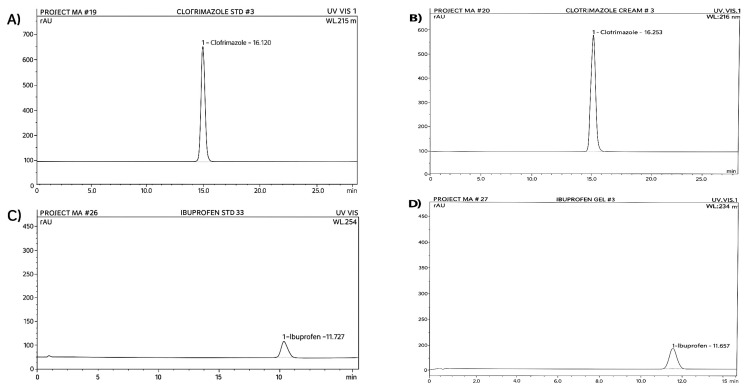
(**A**) Chromatogram of standard clotrimazole; (**B**) chromatogram of clotrimazole cream; (**C**) chromatogram of standard ibuprofen; (**D**) chromatogram of ibuprofen in emulgel, stored under accelerated conditions (40 °C/75% RH) for three months.

The HPLC analysis values of the active ingredients of clotrimazole and ibuprofen over three months were 99.9% and 97.5%, respectively, at day zero, and then decreased to 97.5% and 95.5% during the first month, 96% and 94% during the second month, and finally 95% and 92% during the third month of storage (Figure 2). The results indicate that clotrimazole cream and ibuprofen gel are stable and unaffected by the addition of *P. guajava* leaf extract.

#### 2.4.2. Chemical Stability Test of Pharmaceutical Preparations

The results in Table 8 and Table 9 for preparations stored under accelerated conditions (40 ± 2 °C/75% ± 5% RH) to assess their stability showed that clotrimazole cream and ibuprofen gel, both containing a natural preservative based on 10% *P. guajava* leaf extract powder, have similar characteristics to those containing a chemical preservative, with the exception of color. Although the natural preservative does not affect odor or pH, it induces a change in color into an accepted beige in the cream and changes the texture of the gel to an oily consistency due to its brown tint, respectively, for the clotrimazole cream and the ibuprofen gel. Nevertheless, the final color remains acceptable.

### 2.5. Microbial Stability of Pharmaceutical Preparations

#### 2.5.1. Direct Transfer (Broth Media) for Clotrimazole Cream and Ibuprofen Emulgel

The results presented in Table 10 and Table 11 of the microbial limit tests of clotrimazole cream and ibuprofen gel with 10% *P. guajava* leaf extract showed no microbial growth, comparable to the positive control with chemical preservatives. The natural preservative appears to be effective when compared with the positive control.

However, the negative control showed bacterial growth but no fungal growth for the cream. Regarding ibuprofen gel, the natural and chemical preservatives confirmed the efficacy of the natural preservative. However, the negative control showed bacterial and fungal growth.

#### 2.5.2. Microbial Limit Test—Total Count for Clotrimazole Cream and Ibuprofen Emulgel

The results of the microbial counts of the clotrimazole cream and ibuprofen gel are shown in Table 12 and Table 13. Clotrimazole creams containing 10% *P. guajava* leaf extract and chemical preservatives showed controlled levels of microorganisms, while the negative control revealed high levels of bacteria.

Similarly, in the second formulation of ibuprofen emulgel, emulgels with the same 10% natural preservative showed similar results, with regulated levels of microorganisms, unlike the negative control.

## 3. Discussion

The antimicrobial activity of *Psidium guajava* (L.) has been proven and documented in a number of studies and publications [20,21,22,23], which gives us confidence in the use of this plant as an effective preservative. High levels of important bioactive components were found in the *P. guajava* extract by HPLC quantification. These data indicate higher concentrations than those reported in the literature for guava leaf extracts. Gallic acid was about two times higher than the average (265 mg/100 g) reported by Nguyen et al. [20], and catechin was two times higher than the average reported by Umar et al. [24]. Particularly in functional foods, nutraceuticals, or natural preservatives, the predominance of gallic acid and catechin hydrate suggests possible health-promoting uses.

Overall, the findings indicate that *Psidium guajava* (L.) leaf extract exhibited weak or no antifungal activity in all pharmaceutical products under investigation (ibuprofen gel, clotrimazole cream, permethrin cream, gentamicin cream, and indomethacin emulgel) and strong antibacterial activity against tested bacterial strains. The natural extract’s antibacterial properties were on par with those of artificial preservatives.

Clotrimazole cream and ibuprofen gel, both of which contain a natural preservative based on 10% *P. guajava* leaf extract powder, have similar qualities to those containing a chemical preservative, with the exception of color, according to the results of preparations stored under accelerated conditions (40 ± 2 °C/75% ± 5% RH) to evaluate their stability.

Clotrimazole cream showed satisfactory results, with complete inhibition of microorganism strains such as *Escherichia coli*, *Pseudomonas aeruginosa*, and *Staphylococcus aureus*. For permethrin cream, gentamycin cream, and indomethacin emulgel, the results indicated valid efficacy against all the strains of microorganisms tested, with no increase in the initial number calculated at 14 and 28 days. For ibuprofen gel, complete inhibition was observed for *Escherichia coli* and *Staphylococcus aureus*, with an acceptable reduction in *Pseudomonas aeruginosa*, and no increase from baseline was calculated at 14 and 28 days for *Candida albicans* and *Aspergillus (niger) brasiliensis*. The ibuprofen gel formulation includes propylene glycol and isopropyl alcohol, which may potentiate the efficacy of *P. guajava* extract as a preservative. The preparations that passed the tests, including clotrimazole cream and ibuprofen gel, were subjected to a number of other tests, such as microbial limit tests, drug content determination, all chemical tests, and stability tests, to ensure the safe use of *P. guajava* leaf extract powder as a preservative in pharmaceutical drugs.

In addition, the preparations were tested without preservatives (either chemical or natural) to assess their resistance to contamination. The results confirmed the failure of these preparations and underlined the importance of adding a preservative to prevent contamination. Herman et al. [25] found that the essential oils of *Lavandulla officinallis*, *Melaleuca alternifolia, and Cinnamomum zeylanicum* have higher inhibitory activity against the microbial strains tested than methylparaben. Clotrimazole cream and ibuprofen gel containing 10% *P. guajava* leaf extract showed similarity to those containing chemical preservatives, particularly with regard to odor and pH. However, differences were observed in terms of physical appearance, notably a change in color to an acceptable beige in the case of the cream and an oilier texture for the gel. These changes are attributed to the brown hue of the natural preservative. Stability tests carried out under accelerated conditions confirm these observations. These results were consistent with those of the study by Haque et al. [26]. Microbial limit tests revealed an absence of microbial growth in the pharmaceutical products, clotrimazole cream and ibuprofen gel, containing *P. guajava* leaf extract as a natural preservative, compared to positive controls with chemical preservatives. This efficacy was confirmed in several tests carried out over storage periods of up to three months. HPLC analysis of the active ingredients in clotrimazole cream and ibuprofen gel over three months showed a slight decrease in their concentration, although within acceptable limits, and their stability was not affected by the addition of *P. guajava* leaf extract. These results suggest that natural preservatives based on *P. guajava* leaf extract could be viable and effective alternatives to the chemical preservatives used in pharmaceutical products. Microbial count results for clotrimazole cream revealed controlled levels of microorganisms in both formulations tested: that with 10% *P. guajava* leaf extract and that with a chemical preservative (methyl paraben and propyl paraben). In both cases, bacteria levels were below 10 (limits on agar ≤ 200), as were yeast and mold levels, which were also below 10 (limits on agar ≤ 20). In contrast, the negative control (without preservatives) showed high levels of bacteria compared to the formulations with preservatives. The negative control also showed very high levels of bacteria and fungi compared to formulations with preservatives. These results were slightly similar to those of the study by Al-Rimawia et al. [27], where pharmaceutical formulations containing natural preservatives (olive leaf extract and a mixture of oleuropein and thyme oil) proved stability and effectiveness during three months of storage under accelerated conditions (40 °C/75% RH).

Antibacterial and antifungal stability is greatly increased when 10% *P. guajava* leaf extract is added to clotrimazole cream. Guava leaves’ inherent bioactive substances, particularly their flavonoids, tannins, and essential oils, have broad-spectrum antibacterial properties [28,29].

Adding 10% *Psidium guajava* leaf extract to gentamycin cream improved antibacterial preservation, especially against common skin infections such as *P. aeruginosa*, *E. coli*, and *S. aureus*. The poor antifungal effectiveness, especially against *Aspergillus brasiliensis* and *Candida albicans*, indicates the need for additional antifungal drugs or greater doses. These results lend credence to the use of *P. guajava* extract as a natural antimicrobial preservative in topical preparations, particularly in cases where the main issue is bacterial contamination.

Despite the weak antifungal characteristics of *P. guajava*, the formulation may not have had enough penetration or release, or the concentration (10%) may not have been enough to counteract the significant fungal load [30].

Effective antimicrobial preservation, especially against Gram-positive and Gram-negative bacteria, was achieved by adding 10% *Psidium guajava* leaf extract to Indomethacin emulgel and ibuprofen gel. *S. aureus*, *E. coli*, and *P. aeruginosa* were significantly inhibited by the extract’s bioactive components, which include flavonoids, tannins, and phenolics [28,29]. Higher doses or other antifungal agents could be required for complete preservation efficiency, as the antifungal impact was only weak against *Aspergillus brasiliensis* and moderate against *Candida albicans* [30].

Silva et al. [31] showed that extracts of *P. guajava* exhibited antibacterial activity against bacterial species: *Streptococcus salivarius*, *Streptococcus mutans*, *Streptococcus mitis*, *Streptococcus sanguinis*, and *Streptococcus sobrinus*. Weli et al. [32] studied the antibacterial activity of essential oils derived from *P. guajava* against; *Enterococcus faecalis*; and *Staphylococcus aureus*. Although *P. guajava* leaf extract showed strong antibacterial activity, several formulations showed low antifungal efficiency. These results imply that while these natural preservatives might not be able to fully replace synthetic compounds, they can have a beneficial synergistic effect. Natural substances like guava extract may improve overall antibacterial coverage and lessen the dosage of synthetic agents when used with lower quantities of artificial preservatives. This approach might increase the biocompatibility of the product while reducing the possible negative effects of chemical preservatives.

Propylene glycol and other excipients enhanced the antibacterial properties of guava extract, but they were ineffective as stand-alone preservatives. Coverage was increased to THE USP-mandated spectrum by guava extract, especially against fungi where excipients were ineffective.

The decreased anti-*E. coli* effectiveness of clotrimazole cream may be attributed to result of both pH-mediated solubility restrictions and oil-phase sequestration of guava phenolics. On the other hand, ibuprofen and other hydrophilic gels promoted quick phenolic diffusion to bacterial targets [33].

Clotrimazole and other lipophilic lotions trapped guava phenolics in oil phases, delaying antibacterial activity while extending effectiveness. Rapid phenolic diffusion was made possible by hydrophilic gels (like ibuprofen), but antifungal solubility was restricted [34].

Stronger preservation was required for high-water formulations (such as permethrin cream, which contains 62.2% water) because of the increased microbiological risk.

The lipophilicity of clotrimazole might have enhanced antifungal delivery by co-localizing with guava phenolics in oil phases [35]. The acidic pH of ibuprofen (4.5) decreased phenolic solubility, which hindered the suppression of *Candida albicans*.

Microbial susceptibility and preservative effectiveness are also affected by water content. In our formulations, there were notable differences in water content (ibuprofen gel: approximately 50% (hydrophilic gel); clotrimazole cream, W/O lipophilic: 49.7%; permethrin cream, W/O lipophilic: 62.2%; and gentamicin cream, lipophilic (W/O): about 66%).

Increased water activity that promotes the growth of bacteria and fungi improved diffusion of nutrients in aqueous phases. Risk is decreased by lower-water systems (such as clotrimazole cream, which contains 49.7% water in W/O emulsions) through a discontinuous water phase (microbial access is limited by oil-trapped droplets). Most pathogens are inhibited at lower values (<0.8).

According to our findings, 10% guava extract successfully made up for variations in water content: high-water formulations (permethrin/gentamicin creams) showed complete bacterial inhibition despite having >60% water content (Table 2 and Table 3). This may be attributable to the fact that guava phenolics (e.g., gallic acid) partition into aqueous phases, disrupting microbial membranes [12,14,23]. Lower-water gels (ibuprofen) exhibited stronger bacterial suppression but weaker antifungal action (Table 5), likely due to limited water-soluble antifungal compounds (e.g., flavonoids).

The results of our study indicate that clotrimazole creams had apparent antifungal synergy results from formulation-specific interactions between *P. guajava* extract and the special ingredients of the creams. In this context, gallic acid and other guava phenolics may increase the membrane disruption caused by clotrimazole by increasing the permeability of fungal cells (cell walls are damaged by phenolics). Drug resistance mechanisms are blocked by flavonoids that inhibit efflux pumps. Excipient-Driven Effects Guava extract’s antifungal delivery was especially improved by clotrimazole cream’s lipophilic basis (W/O emulsion). Guava extract and the physicochemical environment interact differently across different formulations, which accounts for the variations in efficiency against *E. coli* [12,14,23].

It was found that bioactivity was dependent on pH 6.0–6.1 for clotrimazole cream (Table 8). Near-neutral pH was preferred: the phenolic acids found in guavas, such as gallic acid (pK_a_~4.5), are stable, which improves their ionization and membrane disruption. The Gram-negative outer membrane of *E. coli* becomes unstable at pH values greater than 5. Ibuprofen gel’s pH ranges from 4.4 to 4.5 (Table 9). An acidic pH could reduce the solubility and absorption of phenolic acids by protonating them in *E. coli*, eliciting acid-stress reactions in bacteria, and boosting resistance [12,14,23].

## 4. Materials and Methods

### 4.1. The Collection of Psidium guajava Leaves and Extraction

In 2023, fresh leaves of Psidium guajava L. were gathered from the Palestinian town of Qalqilya during the blossoming season. The plant material was verified and kept at the Arab American University’s Pharmacology Laboratory. Using 70% ethanol as a solvent, 100 g of dried and crushed leaves were extracted with the use of ultrasonic technology (frequency: 40 kHz; duration: 30 min) to enhance bioactive compound yield. The crude extract was obtained by utilizing an ultrasonic bath at room temperature for 30 min, filtered, concentrated under reduced pressure (40 °C), and lyophilized to obtain a dry powder. The crude extract was then kept at 4 °C until it was needed.

### 4.2. Chemical Characterizations of Psidium guajava (L.) Leaf Extract Using High-Performance Liquid Chromatography (HPLC)

HPLC-grade methanol (A) and purified water (B) with 1.0% acetic acid (*v*/*v*) were the analytical solvents used in the liquid chromatographic separation, which was carried out at 40 °C. A Purospher^®^ RP-18 Endcapped column (5 µm, 250 mm length; Merck KGaA, Darmstadt, Germany) was used in the HPLC procedure. Chromatography was carried out at a steady flow rate of 1.0 mL/min for 45 min. The gradient program was 100% B for 0–10 min, 70% B for 10–20 min, 10% B for 20–30 min, 70% B for 30–37 min, and 100% B for 37–45 min. The wavelength at which UV chromatograms were obtained was 280 nm.

About 0.6 g of the extracted powder was transferred to a volumetric flask in order to prepare the HPLC sample. After adding 90 mL of ethanol, the liquid was agitated for half an hour. Prior to HPLC and spectroscopic examination, the solution was mixed and filtered through a PTFE syringe filter (0.45 µm) after purified water was added to adjust the volume. By contrasting the retention durations and spectra with those of established standards, identification and qualitative analysis were carried out [36]. Gallic acid (C_7_H_6_O_5_), catechin (C_15_H_14_O_6_), chlorogenic acid (C_16_H_18_O_9_), caffeic acid (C_9_H_8_O_4_), and rutin hydrate (C_27_H_32_O_17_) were among the chemicals found at 280 nm (Figure S1).

### 4.3. Preparation of Semi-Solid Dosage Forms Containing Psidium guajava (L.) Leaf Extract

#### 4.3.1. Preparation of Ibuprofen Gel

This pharmaceutical preparation was designed using the protocol established by Kashyap et al. [33], with a specific adaptation for our research. The new gel formulation was developed by using ibuprofen (as a non-steroidal anti-inflammatory drug) (5.0% *w*/*w*) as the active molecule with 23 g isopropyl alcohol (solvent). The ingredients were mixed in a container for 5 min using a hand blender; this mixture was transferred to another container containing 20 g propylene glycol (solvent) and mixed for 5 min. Then, a solution containing 10 g *P. guajava* leaf powder in 38 g pure water was added while stirring continuously, followed by the addition of 3 g Sepigel (viscosity increasing agent) and 1 g isopropyl myristate (vehicle). The formulation was mixed for 5 min using a hand mixer. Three preparations were developed for this formulation, the first of which included a natural preservative based on an extract of the plant studied, at a concentration of 10%. The second preparation, used as a positive control, was formulated with chemical preservatives, notably 0.12% methyl and 0.08% propyl paraben. The third preparation, used as a negative control, was preservative-free.

#### 4.3.2. Preparation of Clotrimazole Cream

Clotrimazole cream was prepared according to the protocol of Shahin et al. [34], modified according to the needs of our study. The cream preparation method is based on the use of clotrimazole (which is often used as an antifungal agent for intravaginal application) 1% (*w*/*w*) as the active molecule in this formulation, which begins by transferring 13 g of white petrolatum (emollient), 10.5 g of paraffin oil, 8.80 g of lanette O (emulsifying agent), and 1.69 g of Emulgin B2 (emulsifying agent) into mixing vessel N°1, which was heated to a temperature of 80–85 °C (oil phase). Clotrimazole was then added to the oil phase in mixing vessel N°1. The aqueous phase was prepared in mixing vessel N°2 by mixing 4.31 g glycerin (emollient) and the solution containing 10 g *P. guajava* leaf powder with 49.7 g pure water (solvent) (80 and 85 °C), then mixing for 2 min. The aqueous phase was transferred from vessel no. 2 to mixing vessel no. 1 and then cooled to 35 °C while mixing. Three preparations were developed for this formulation, the first of which included a natural preservative based on an extract of the plant studied, at a concentration of 10%. The second preparation containing chemical preservatives (notably 0.12% methyl and 0.08% propyl paraben) was used as a positive control. The third preparation, used as a negative control, was preservative-free.

#### 4.3.3. Preparation of Permethrin Cream

This formulation consists of 6.49 g Lanolin O (emulsifying), 1.07 g Emulgin B2 (emulsifying), and 1.80 g Lanolin anhydrous (Emulsifying). The ingredients were transferred to container N°1 and then heated to 80–85 °C (oil phase). In vessel N°2, 4.49 g of Crystalline Oil 9 (Oleaginous Vehicle) was heated to about 50 °C; then, 5 g of Permethrin (Scabicide) was added to Crystalline Oil 9 and stirred for about 2 min until dissolved, and the mixture (Permethrin/Crystalline Oil 9) was added to vessel N°1 and stirred for 5 min. To prepare the aqueous phase, 8.95 g of glycerin (emollient) was added to container N°3 and mixed for 3 min using the hand mixer with the solution containing 10 g of *P. guajava* leaf extract prepared in 62.2 g of pure water. This aqueous phase was gradually added from mixing vessel N°3 into the mixture in vessel N°1, followed by mixing for 5 min, and was cooled until the temperature reached 35 °C. Three preparations were developed for this formulation, the first of which included a natural preservative based on an extract of the plant studied, at a concentration of 10%. The second preparation, used as a positive control, was formulated with chemical preservatives, notably 0.12% methyl and 0.08% propyl paraben. The third preparation, used as a negative control, was preservative-free.

#### 4.3.4. Preparation of Gentamicin Cream

Gentamicin cream was prepared according to Pick et al. [35], with a modification adapted to our study. To prepare this formulation, 6.79% Lanette O (emulsifying agent), 1.7% Emulin B2 (emulsifying agent), 14.1% white petrolatum (Oil Phase), and 7.9% Crystal Oil 9 (Oil Phase) were transferred to vessel N°1 and then heated to 80–85 °C (oil phase). Then, 5% of the total amount of purified water in vessel N°2 was heated to 45 °C. The aqueous phase was prepared by mixing the purified water (solvent) with 10% *P. guajava* leaf powder in vessel N°3 (80–85 °C), gradually adding the aqueous phase from mixing vessel N°3 to the mixture in vessel N°1, then heating at 45 °C for 5 min, followed by transferring the mixture of 0.1% gentamicin (Aminoglycoside antibiotic) from mixing vessel N°2 to mixing vessel N°1, mixing for 5 min with the hand mixer, and then cooling while mixing until the temperature reached 35 °C. Three preparations were developed for this formulation, the first of which included a natural preservative based on an extract of the plant studied, at a concentration of 10%. The second preparation, used as a positive control, was formulated with chemical preservatives, notably 0.12% methyl and 0.08% propyl paraben. The third preparation, used as a negative control, was free of preservatives.

#### 4.3.5. Preparation of Indomethacin Emulgel

Indomethacin emulgel was developed following the protocol of Kaleemullah et al. [37], with a specific adaptation integrated into our study.

In container N°1, 1 g of indomethacin was dissolved in 10 g of medium-chain triglycerides (MCT oil) at 45 °C to create the oil phase. In container N°2, 10% *P. guajava* leaf extract and 50% purified water were present in the aqueous phase. The gelling/emulsifying agent Sepineo^®^ P600 was used to emulsify the two phases. The preparation of this pharmaceutical product was formulated by adding 1 g of indomethacin (anti-inflammatory, analgesic) to container N°1, which contained 50% purified water (solvent), and container N°2, which contained 50% purified water with 10% *P. guajava* leaf powder (antimicrobial preservative), and mixing for 10 min using the hand blender, then transferring to container N°1 while stirring for 3 min. Then, ethanol (solvent) was added and mixed for 10 min using a hand blender, and 1 g of glycerin (emollient) was added to the container and mixed for 2 min. In total, 3 g of Sepineo P600 (gelling Agent) was added to the container and mixed for 5 min while stirring. Three preparations were developed for this formulation, the first of which included a natural preservative based on an extract of the plant studied, at a concentration of 10%. The second preparation, used as a positive control, was formulated with chemical preservatives, notably 0.12% methyl and 0.08% propyl paraben. The third preparation, used as a negative control, was preservative-free.

### 4.4. Chemical and Physical Analytical Tests

#### 4.4.1. Test for Ibuprofen Gel, Clotrimazole Cream and Permethrin Cream

The active ingredients that were used in the five formulations were identified and quantified by high-performance liquid chromatography (HPLC), using a mobile phase made up of different solvents. According to ICH Q2(R1), the specificity, linearity, accuracy, precision, and robustness of HPLC techniques were validated. The system appropriateness requirements were met in every analysis.

For ibuprofen gel, clotrimazole cream, and permethrin cream, the mobile phases varied, but they were generally made up of glacial acetic acid, acetonitrile, methanol, and a specific buffer. The mobile phase used for ibuprofen comprised 62% glacial acetic acid and 38% acetonitrile, for clotrimazole cream, it comprised 80% methanol and 20% buffer (containing 4.35 g dibasic potassium phosphate in water to obtain a 1000 mL solution), and for permethrin cream, it comprised 83% methanol (MeOH) and 17% buffer (0.5% acetic acid in 1000 mL purified water). The stationary phase for ibuprofen included a 125 × 4 mm, 5 µm particle size Lichrospher RP-Select B column, for clotrimazole cream a 25 cm × 4.6 mm RP-18e (5 µm) column, and for permethrin cream a 125 × 4 mm, 5 μm particle size Lichrospher RP-18e column. The stationary phase flow rate was 1.5 mL/min for all samples, except for clotrimazole cream, which had a flow rate of 1.2 mL/min. The volume of injected sample was set to 20 µL, with detection performed at a wavelength of 254 nm for ibuprofen and clotrimazole, while permethrin cream was detected at 240 nm. Quantification of the active ingredients in these formulations was performed using peak area and calibration curves obtained from standard solutions of ibuprofen, clotrimazole, and permethrin. Identification was carried out according to these protocols, with slight modifications [38,39,40].

#### 4.4.2. Test for Gentamicin Cream

In this protocol, for the biological determination of gentamicin sulfate, preparation of the standard solution and sample involved several steps. Firstly, a precisely weighed quantity of the USP Reference Standard (RS) or Working Standard of the antibiotic under investigation, ranging from 12.5 to 100 mg, was dissolved to form a stock solution at a specified concentration. This stock solution was then diluted in a specified solvent to reach the required target concentrations. The prepared standard solution was stored under appropriate conditions, such as refrigeration, if necessary and used within the recommended timeframe, as specified in the validation method for each antibiotic. At the same time, the unknown sample was assigned an estimated potency per unit weight or volume, based on the potency declared by the manufacturer. On the day of the assay, a stock solution and a test solution for the unknown sample were prepared using the same final diluent as that used for the standard solution. From the sample stock solution, three target concentrations of the test sample, as well as standard concentrations, were prepared, covering a range from lowest to highest.

#### 4.4.3. Chemical Stability Testing of Pharmaceutical Preparations

Clotrimazole cream and ibuprofen gel were tested for chemical stability over a three-month period under accelerated conditions of 40 °C/75% RH. Several characteristics were evaluated to ensure product quality and stability. Firstly, the pH of the formulations was measured using a pH meter (Mettler Toledo S220-Kit, Greifensee, Switzerland). The viscosity of the formulations was assessed by taking 50 mL of the preparation in a 50 mL beaker and then using an N 4 spindle at 100 rpm at a controlled temperature between 20 and 22 °C, with the use of a Brookfield DV2T viscometer (Middleboro, MA, USA). Finally, the density of the formulations was determined using a pycnometer. These tests were carried out according to rigorous standards to guarantee the quality and efficacy of the products throughout their shelf life [41,42].

### 4.5. Testing the Effectiveness of Antimicrobial Preservative (Protection)

#### 4.5.1. Tested Microorganisms

The microorganisms used in our study were stored in the facilities of the Pharmacology and Phytomedicine Laboratory of the Arab American University (Jenin, Palestine). They included three bacterial strains, Gram-positive *S. aureus* (ATCC 6538), Gram-negative *E. coli* (ATCC 8739), and *P. aeruginosa* (ATCC 9027), as well as two fungal strains: *C. albicans* (ATCC 10231) as a yeast and *A. brasiliensis* (ATCC 16404) as a mold. All these bacterial and fungal strains were sub-cultured from the original freeze-dried culture, which had undergone no more than five passages in culture since the initial freeze-drying [43].

The procedure starts by rinsing the growth surface with sterile saline solution, followed by collection in a suitable container and then addition of an appropriate amount of sterile saline solution to achieve a microbial density of around 1 × 10^8^ CFU/mL. Cells are quantified by measuring turbidity with a spectrophotometer at a wavelength of 650 nm to obtain specific optical densities: A. 0.3–0.45 for *S. aureus* (~1–3 × 10^8^ CFU), B. 0.2–0.3 for *P. aeruginosa* and *E. coli* (~1–3 × 10^8^ CFU), and C. < 1.0 for *C. albicans* (~1–3 × 10^8^ CFU). Various dilutions are prepared (10^−3^–10^−6^) and inoculated using the plate count method (1 mL of each dilution in duplicate in Sabouraud Dextrose agar). Cultures are then incubated for 24 h at 35 °C ± 2 for bacteria, and at 23 °C ± 2 for 2–3 days for *Candida albicans* fungi. Finally, CFUs are counted from counting plates containing between 30 and 100 CFUs [44].

The fungal growth on the surface of media was rinsed with a sterile saline solution containing 0.05% polysorbate 80. A sufficient quantity of sterile saline was then added to obtain a microbial concentration of around 1 × 10^8^ CFU per ml. Successive dilutions (from 10^−3^ to 10^−8^) were then made and inoculated using the pour plate method (1 mL of each dilution in Sabouraud Dextrose agar, in duplicate). Plates were incubated for 2 to 4 days at 23 ± 2 °C, after which CFUs were counted (counting plates containing between 10 and 100 CFUs). Organisms from the mother culture can be grown in a suitable liquid medium such as soy casein digestion broth or Sabouraud dextrose broth. The cells were harvested by centrifugation, washed, and resuspended in sterile TS saline to obtain a microbial concentration of around 1 × 10^8^ CFU/mL. The microbial load in each suspension was determined using the media conditions and incubation times for microbial recovery to confirm the initial estimate of CFU per ml [25].

#### 4.5.2. Evaluation of the Effectiveness of Preservation of Prepared Formulas

The experimental protocol was conducted separately for each strain in five sterile bacteriological containers. Each container was filled with a 20 mL sample of the prepared formulations, namely clotrimazole cream, permethrin cream, gentamycin cream, ibuprofen gel, and indomethacin gel, each containing 10% *w*/*w Psidium guajava* (L.) extract. Each container was then inoculated with a prepared standardized inoculum. The volume of inoculum in the suspension varied between 0.5% and 1% of the product volume. To achieve a target concentration in the test preparation after inoculation of between 1 × 10^5^ and 1 × 10^6^ CFU per mL of product, the concentration of test microorganisms that was added to the product was adjusted accordingly. For example, if we inoculated 100 to 200 µL of a 1 × 10^8^ CFU/mL inoculum into a 20 mL sample, the final concentration would be 0.5 to 1 × 10^6^ CFU/mL. Inoculated containers were incubated at 23 ± 2 °C. Samples were taken from each vessel at specific time intervals: 0, 7, 14, and 28 days.

According to standard antimicrobial preservative testing protocols, the concentration of microbial suspensions used to challenge the formulations was adjusted to achieve 10^6^ CFU/mL for bacterial strains (*Staphylococcus aureus*, *Escherichia coli*, and *Pseudomonas aeruginosa*) and 10^5^ CFU/mL for fungal strains (*Candida albicans* and *Aspergillus brasiliensis*).

Successive 1:10 dilutions were made by taking 1 mL of each container, containing the microorganism to be tested, and transferring them to test tubes containing 9 mL of Letheen broth medium, to obtain dilutions of 10^−1^ to 10^−5^ depending on experimental conditions. Next, 1 mL of each dilution of each microorganism was pipetted and transferred to sterile 90 mm Petri dishes. Subsequently, 15 mL of Letheen’s agar medium was poured into each plate at a temperature of 48 °C. Plates were left to solidify under a fume hood; then, bacterial plates were incubated at 35 ± 2 °C for 2–3 days, and fungal plates were incubated at 23 ± 2 °C for 5–7 days. Colony-forming units (CFUs) were counted by the plate counting method for each preparation tested at specific time intervals. Using the initial CFU/mL concentrations in the suspension, the change in log10 values of CFU/mL concentration for each microorganism at the appropriate test intervals was calculated, and changes were expressed in terms of log reduction, defined as the difference between the log value of the initial CFU/mL concentration in the suspension and the log value of CFU/mL of the survivors at that time.

The natural preservative (*Psidium guajava* extract) and other active pharmaceutical ingredients (clotrimazole, permethrin, gentamicin, ibuprofen, and indomethacin) in the formulations, as well as possible antimicrobial enhancers like EDTA, were all rendered inactive by the neutralizing medium of Letheen broth. To verify that Letheen broth is efficient in promoting the recovery of challenged bacteria, a neutralization validation test was conducted.

The direct transfer test’s goal was as follows:

The pharmaceutical formulations’ microbiological stability during accelerated storage was evaluated using the antimicrobial limit (direct transfer) test. This technique helps ascertain if the formulations retain sterility or regulated microbial levels without purposeful inoculation, in contrast to challenge testing. In order to guarantee that the added preservative, *Psidium guajava* leaf extract, efficiently prevents any microbial contamination that could happen during handling, storage, or repeated usage, it replicates real-world circumstances.

#### 4.5.3. Antimicrobial Effectiveness Test

Antimicrobial efficacy criteria for topical products are established according to specific standards. For bacteria, a reduction of at least 2.0 logs compared to the initial count must be observed after a period of 14 days. As for yeasts and molds, no increase must be observed compared to the initial count, assessed after 14 and 28 days, respectively.

In compliance with USP <51>, which calls for a minimum of four sample points during the 28-day testing period, microbial counts were assessed on Days 0, 7, 14, and 28. The present investigation adhered to the USP timetable, even though the British Pharmacopeia (BP) suggests a follow-up assessment on Day 21. This is seen as a barrier to further standardization.

The USP <51> acceptance criteria were applied to assess the efficacy of the preservative:

1. A ≥ 2.0 log_10_ decrease in the bacterial count by Day 14.
2. No increase in the bacterial count after Day 14.
3. No increase in the fungal count (yeasts and molds) from the initial inoculum during the test period

To ascertain preservative efficacy, these standards were applied to every formulation that was tested.

#### 4.5.4. Aerobic Microbial Count (AMC) and Total Yeast and Mold Count (TYMC)

This protocol aims to qualitatively and quantitatively assess total viable microorganisms (TAMC) and total yeasts and molds (TYMC) in the finished products, such as creams and gels containing our plant extract, following USP and BP pharmaceutical quality standards. To prepare samples, tubes and caps were carefully disinfected with 70% ethanol, and then the products were transferred to sterile vials before being heated to 45 °C to obtain a uniform suspension. The vials were placed under a laminar flow hood to maintain a sterile environment for subsequent handling. The direct transfer and plate counting method for bacterial counts involved transferring 10 mL of dissolved sample to a vial and then adding 90 mL of selective culture medium to obtain a final dilution of 1:10. One ml of this dilution was transferred to a Petri dish containing solid or liquid culture medium and incubated at 30–35 °C for 3–5 days. The results were then observed and expressed as the average number of microorganisms per gram or milliliter. For the total count of aerobic yeasts and molds, the same method was used, replacing the culture medium with Sabouraud Dextrose Agar and Borth (SADM); then, the plates were incubated at 20–25 °C for 5–7 days [25].

#### 4.5.5. Acceptance Criteria for Finished Products

According to USP/BP standards for the cutaneous route of administration, the total number of aerobes on solid media (TAMC) must be less than 200 colony-forming units per gram (<200 CFU/g), with a result of 10^2^. Similarly, the total number of yeasts and molds on solid media (TYMC) must be less than 20 colony-forming units per gram (<20 CFU/g), with a specific result of 10^1^. In addition, no detectable presence of *Staphylococcus aureus* or *Pseudomonas aeruginosa* is permitted. The interpretation of USP/BP acceptance criteria is as follows:

For 10^1^ CFU, the maximum acceptable number is 20.

For 10^2^ CFU, the maximum acceptable number is 200.

### 4.6. Stability Test

Using 10% *P. guajava* leaf extract as a natural preservative, formulations of ibuprofen gel, clotrimazole cream, permethrin cream, gentamycin cream, and indomethacin emulgel received accelerated stability testing for three months at 40 ± 2 °C and 75% ± 5% relative humidity (RH). The United States Pharmacopeia (USP) recommendations were followed in the implementation of this procedure, which is in accordance with the ICH Q1A(R2) stability requirements that are acknowledged by major pharmacopeias, such as the European Pharmacopeia (EP) and the British Pharmacopeia (BP).

The goal was to evaluate the formulations’ stability in terms of their physical, chemical, and microbiological properties under stress that simulates long-term storage. The following were among the parameters assessed:

- Chemical analysis of the content of active ingredients (by HPLC);
- Physical attributes, such as color, texture, and smell, and measurement of pH;
- Using microbiological limit tests and preservative challenges, antimicrobial efficacy.

## 5. Conclusions

The novelty of this research lies in the use, for the first time, of the *P. guajava* plant as a natural preservative in five pharmaceutical preparations at a concentration of 10% *w*/*w*, instead of using a chemical preservative. The results of our study on *P. guajava* leaf extract show that of the five preparations tested, two passed all the preservation tests. However, the effectiveness of this plant extract as a preservative in these pharmaceutical preparations is limited, as chemical preservatives demonstrated an observable priority over it, even though it was close to their results. Our study also makes a significant contribution by enabling researchers to further explore the potential of plant molecules as preservatives in pharmaceutical formulations. It also paves the way for further formulation optimization experiments. Guava’s antimicrobial chemicals’ formulation-specific bioavailability, which is influenced by pH, excipients, and API interactions, is the cause of the varied efficacy. This emphasizes the necessity of optimizing formulations when utilizing natural preservatives.

Several approaches have been put forth in the literature to address the limitations of some natural preservatives’ weak antifungal efficacy. These include (1) synergistic combinations of natural extracts with chemical preservatives at lower concentrations; (2) encapsulation techniques that enhance the bioavailability of preservatives; and (3) optimized formulation designs that improve the release or penetration of bioactive components. To increase its range of action and lessen dependency on artificial substances, future research may investigate mixing *P. guajava* with additional antibacterial agents.

## Figures and Tables

**Table 1:** Preservative efficacy test of clotrimazole cream containing 10% *P. guajava* leaf extract powder as a natural preservative.

Microorganism 10^6^ CFU/mL	ATCC No.	Day	Tested Product with 10% Extract	Control
*S. aureus*	6538	0	4.70	6.78
		7	3.53	>9
		14	<1	>9
		28	<1	7.87
*P. aeruginosa*	9027	0	6.06	7.32
		7	3.77	>9
		14	<1	>9
		28	<1	>9
*E. coli*	8739	0	7.45	7.32
		7	5.04	>9
		14	4.04	>9
		28	2.98	>9
*Candida albicans*	10231	0	4.69	4.08
		7	<1	3.67
		14	<1	6.95
		28	<1	8.68
*A. brasiliensis*	16404	0	4.67	4.84
		7	4.73	6.83
		14	<1	<1
		28	<1	<1

Test of the preservation efficiency of clotrimazole cream with 10% *P. guajava* extract. Measuring unit: Log CFU/mL (mean, *n* = 3) is shown by the data. Controls: formulation without preservatives. As per USP <51>, acceptance was as follows: Day 14: bacterial decrease ≥ 2-log; and Day 28: no fungal increase.

**Table 2:** Preservative efficacy test of permethrin cream containing 10% *P. guajava* leaf extract powder as a natural preservative.

Microorganism 10^6^ CFU/mL	ATCC No.	Day	Tested Product with 10% Extract	Control
*S. aureus*	6538	0	7.05	6.83
		7	<1	>9
		14	<1	>9
		28	<1	7.04
*P. aeruginosa*	9027	0	5.74	6.63
		7	4.32	>9
		14	<1	>9
		28	<1	8.04
*E. coli*	8739	0	6.4	6.79
		7	3.85	>9
		14	<1	>9
		28	<1	>9
*Candida albicans*	10231	0	7.12	5.09
		7	7.11	6.83
		14	6.83	6.9
		28	6.04	6.57
*A. brasiliensis*	16404	0	5.05	5.2
		7	5	4.92
		14	4.99	4.56
		28	4.51	5

Test of the preservation efficiency of clotrimazole cream with 10% *P. guajava* extract. Measuring unit: Log CFU/mL (mean, *n* = 3) is shown by the data. Controls: formulation without preservatives. As per USP <51>, acceptance was as follows: Day 14: bacterial decrease ≥ 2-log; and Day 28: no fungal increase.

**Table 3:** Preservative efficacy test of gentamycin cream containing 10% *P. guajava* leaf extract powder as a natural preservative.

Microorganism 10^6^ CFU/mL	ATCC No.	Day	Tested Product with 10% Extract	Control
*S. aureus*	6538	0	6.25	7.81
		7	<1	>8
		14	<1	>8
		28	<1	>8
*P. aeruginosa*	9027	0	5	6.9
		7	<1	>8
		14	<1	>8
		28	<1	>9
*E. coli*	8739	0	4	7.39
		7	<1	>9
		14	<1	>9
		28	<1	>9
*Candida albicans*	10231	0	7.71	5.56
		7	7.68	6.11
		14	7.47	6.9
		28	7.02	6.53
*A. brasiliensis*	16404	0	6.04	6.17
		7	6	6.39
		14	5.99	6.15
		28	5.81	5.95

Test of the preservation efficiency of clotrimazole cream with 10% *P. guajava* extract. Measuring unit: Log CFU/mL (mean, *n* = 3) is shown by the data. Controls: formulation without preservatives. As per USP <51>, acceptance was as follows: Day 14: bacterial decrease ≥ 2-log; and Day 28: no fungal increase.

**Table 4:** Preservative efficacy test of indomethacin emulgel containing 10% *P. guajava* leaf extract powder as a natural preservative.

Microorganism 10^6^ CFU/mL	ATCC No.	Day	Tested Product with 10% Extract	Control
*S. aureus*	6538	0	4.47	>9
		7	3.98	>9
		14	<1	>9
		28	<1	>9
*P. aeruginosa*	9027	0	3.56	6.78
		7	2.68	>9
		14	<1	>9
		28	<1	>9
*E. coli*	8739	0	3.05	6.79
		7	<1	>9
		14	<1	>9
		28	<1	>9
*Candida albicans*	10231	0	4.74	5.56
		7	5.49	6.3
		14	4.9	6.81
		28	4.4	6.53
*A. brasiliensis*	16404	0	7.9	5
		7	6.76	6
		14	6.65	6
		28	6.08	6.32

Test of the preservation efficiency of clotrimazole cream with 10% *P. guajava* extract. Measuring unit: Log CFU/mL (mean, *n* = 3) is shown by the data. Controls: formulation without preservatives. As per USP <51>, acceptance was as follows: Day 14: bacterial decrease ≥ 2-log; and Day 28: no fungal increase.

**Table 5:** Preservative efficacy test of ibuprofen gel containing 10% *P. guajava* leaf extract powder as a natural preservative.

Microorganism 10^6^ CFU/mL	No.	Day	Tested Product with 10% Extract	Control
*S. aureus*	6538	0	7	>8
		7	<1	8.31
		14	<1	7.91
		28	<1	7.88
*P. aeruginosa*	9027	0	6.99	7.15
		7	5.47	>9
		14	5.04	>9
		28	3.81	>9
*E. coli*	8739	0	6.30	6.79
		7	<1	>9
		14	<1	>9
		28	<1	>9
*Candida albicans*	10231	0	5.85	6.54
		7	5.12	6.8
		14	5.1	7.17
		28	5.05	7.12
*A. brasiliensis*	16404	0	6.05	6.54
		7	6.11	6.04
		14	6.07	7.08
		28	5.94	7.98

Test of the preservation efficiency of clotrimazole cream with 10% *P. guajava* extract. Measuring unit: Log CFU/mL (mean, *n* = 3) is shown by the data. Controls: formulation without preservatives. As per USP <51>, acceptance was as follows: Day 14: bacterial decrease ≥ 2-log; and Day 28: no fungal increase.

**Table 6:** Anti-microbial test for clotrimazole cream with natural preservative, chemical preservative, or no preservative.

Microorganism 10^6^ CFU/mL	ATCC No.	Day	With 10% Extract as a Preservative	Positive Control (Chemical Preservative) %	Negative Control (Without Preservative)	Control Dilution
*S. aureus*	6538	0	5.7	>4	6.77	>9
		7	2.44	<1	7.99	>9
		14	<1	<1	7.56	>9
		28	<1	<1	>9	>9
*P. aeruginosa*	9027	0	6.2	>4	6.77	>9
		7	3.77	<1	>9	>9
		14	<1	<1	7.93	>9
		28	<1	<1	7.6	>9
*E. coli*	8739	0	6.45	>4	7.04	>9
		7	5.9	<1	7.97	>9
		14	3.78	<1	7.93	>9
		28	2.98	<1	7.95	7.67
*Candida albicans*	10231	0	>4	>4	5.77	5.3
		7	<1	<1	5.3	5.7
		14	<1	<1	3.05	6.1
		28	<1	<1	2.34	7.67
*A. brasiliensis*	16404	0	>4	>4	>4	5.3
		7	3.81	<1	>4	5.7
		14	<1	<1	3.05	6.1
		28	<1	<1	2.34	7.67

Measuring unit: Log CFU/mL (mean, *n* = 3) is shown by the data. Controls: formulation without preservatives. As per USP <51>, acceptance was as follows: Day 14: bacterial decrease ≥ 2-log; and Day 28: no fungal increase.

**Table 7:** Antimicrobial testing for ibuprofen gel with natural preservative, chemical preservative, or no preservative.

Microorganism 10^6^ CFU/mL	No.	Day	With 10% Extract as a Preservative	Positive Control (Chemical Preservative) %	Negative Control (Without Preservative)	Control Dilution
*S. aureus*	6538	0	>4	>4	7.16	5.7
		7	<1	<1	7.51	7.31
		14	<1	<1	6.39	6.91
		28	<1	<1	6.08	3.88
*P. aeruginosa*	9027	0	6.99	>4	6.78	>9
		7	3.78	<1	7.99	>9
		14	<1	<1	7.79	>9
		28	<1	<1	7.41	>9
*E. coli*	8739	0	>4	>4	7.04	>9
		7	<1	<1	7.65	>9
		14	<1	<1	7.92	7.91
		28	<1	<1	7.04	7.87
*Candida albicans*	10231	0	6.25	>4	5.78	4
		7	6.04	<1	7.27	2.8
		14	5.95	<1	7.39	3.18
		28	4	<1	7.19	3.12
*A. brasiliensis*	16404	0	6.51	>4	6.21	5.47
		7	6.34	2.94	7.04	6.04
		14	6.28	<1	7.71	7.07
		28	6.15	<1	7.79	7.97

Measuring unit: Log CFU/mL (mean, *n* = 3) is shown by the data. Controls: formulation without preservatives. As per USP <51>, acceptance was as follows: Day 14: bacterial decrease ≥ 2-log; and Day 28: no fungal increase.

**Table 8:** Finished preparation specifications for clotrimazole cream under accelerated conditions (40 °C/75% RH).

Time	Physical Appearance	Odor	PH	Assay of Clotrimazole
Zero time	Beige, thick homogeneous cream	Odorless	6.1	99.9%
First month	Beige, thick homogeneous cream	Odorless	6.0	97.5%
Second month	Beige, thick homogeneous cream	Odorless	6.0	96%
Third month	Beige, thick homogeneous cream	Odorless	6.0	95%

**Table 9:** Finished preparation specifications for ibuprofen gel under accelerated conditions (40 °C/75% RH)

Time	Physical Appearance	Odor	PH	Assay of Ibuprofen
Zero time	Brown (oily), thick consistency Emulgel, no crystals appear	Isopropyl alcohol odor	4.5	97.5%
First month	Brown (oily), thick consistency Emulgel, no crystals appear	Isopropyl alcohol odor	4.4	95.5%
Second month	Brown (oily), thick consistency Emulgel, no crystals appear	Isopropyl alcohol odor	4.4	94%
Third month	Brown (oily), thick consistency Emulgel, no crystals appear	Isopropyl alcohol odor	4.4	92%

**Table 10:** Antimicrobial limit (direct transfer test) of clotrimazole cream under accelerated conditions (40 °C/75% RH).

Time	Direct Transfer (Broth Media)	With 10% *P. guajava* Leaf Extract as a Natural Preservative	Positive Control with Chemical Preservative (Methyl and Propyl Paraben	Negative Control (Without Preservative)
Zero time	Tryptic soy broth	Clear	Clear	Turbid
	Sabouraud dextrose broth	Clear	Clear	Clear
First month	Tryptic soy broth	Clear	Clear	Turbid
	Sabouraud dextrose broth	Clear	Clear	Clear
Second month	Tryptic soy broth	Clear	Clear	Turbid
	Sabouraud dextrose broth	Clear	Clear	Clear
Third month	Tryptic soy broth	Clear	Clear	Turbid
	Sabouraud dextrose broth	Clear	Clear	Clear

**Table 11:** Antimicrobial limit (direct transfer test) of ibuprofen gel under accelerated conditions (40 °C/75% RH)

Time	Direct Transfer (Broth Media)	With 10 % *P. guajava* Leaf Extract as a Natural Preservative	Positive Control with Chemical Preservative (Methyl and Propyl Paraben	Negative Control (Without Preservative)
Zero time	Tryptic soy broth	Clear	Clear	Turbid
	Sabouraud dextrose broth	Clear	Clear	Turbid
First month	Tryptic soy broth	Clear	Clear	Turbid
	Sabouraud dextrose broth	Clear	Clear	Turbid
Second month	Tryptic soy broth	Clear	Clear	Turbid
	Sabouraud dextrose broth	Clear	Clear	Turbid
Third month	Tryptic soy broth	Clear	Clear	Turbid
	Sabouraud dextrose broth	Clear	Clear	Turbid

**Table 12:** Antimicrobial limit (total count test) of clotrimazole cream under accelerated conditions (40 °C/75% RH).

Time	Total Count	With 10 % *P. guajava* Leaf Extract as a Natural Preservative	Positive Control with Chemical Preservative (Methyl and Propyl Paraben	Negative Control (Without Preservative)
Zero time	Tryptic soy agar	<10	<10	<30
	Sabouraud dextrose agar	<10	<10	<10
First month	Tryptic soy agar	<10	<10	<30
	Sabouraud dextrose agar	<10	<10	<10
Second month	Tryptic soy agar	<10	<10	<30
	Sabouraud dextrose agar	<10	<10	<10
Third month	Tryptic soy agar	<10	<10	<180
	Sabouraud dextrose agar	<10	<10	<10

**Table 13:** Antimicrobial limit (total count test) of ibuprofen gel under accelerated conditions (40 °C/75% RH).

Time	Total Count	With 10 % *P. guajava* Leaf Extract as a Natural Preservative	Positive Control with Chemical Preservative (Methyl and Propyl Paraben	Negative Control (Without Preservative)
Zero time	Tryptic soy agar	<10	<10	<100
	Sabouraud dextrose agar	<10	<10	<20
First month	Tryptic soy agar	<10	<10	<100
	Sabouraud dextrose agar	<10	<10	<20
Second month	Tryptic soy agar	<10	<10	<110
	Sabouraud dextrose agar	<10	<10	<20
Third month	Tryptic soy agar	<10	<10	<220
	Sabouraud dextrose agar	<10	<10	<20

## Data Availability

Data are contained within the article.

## Supplementary Materials

The following supporting information can be downloaded at: https://www.mdpi.com/article/10.3390/antibiotics14121176/s1, Figure S1: (A) Chromatograms of standard phenolic substances at 280 nm wavelengths 1—gallic acid (RT = 6.523); 2—catechin (RT = 9.725); 3—chlorogenic acid (RT = 10.624); 4—caffeic acid (RT = 11.731); and 5—rutin (RT = 13.524). (B) HPLC chromatogram of ethanol extract of guava leaves. Peaks: 1—gallic acid (RT = 6.613); 2—catechin (RT = 9.614); 3—chlorogenic acid (RT = 10.611); 4—caffeic acid (RT = 11.491); and 6—rutin (RT = 13.615); (peak 5, 7, 8, and 9 are unknown).

## Abbreviations

The following abbreviations are used in this manuscript:

RH	Relative humidity
HPLC	High-performance liquid chromatography
ATCC	American Type Culture Collection
O.D.	Optical density
CFUs	Colony-forming units
SCDM Digest Medium	Soyabean Casein
CDSLP	Casein digest–soy lecithin polysorbate
FLM	Fluid Lactose Medium
SCDA	Soyabean Casein Digest Agar
