# Supplementary material for: Guava Leaf Extract as a Sustainable Preservative Alternative in Semi-Solid Pharmaceuticals: Efficacy and Stability Assessment
Source: Antibiotics (Basel). 2025 Nov 21;14(12):1176. doi: 10.3390/antibiotics14121176 (PMC12730129; doi:10.3390/antibiotics14121176)
Supplement: Supplementary file 1 [file antibiotics-14-01176-s001.zip › antibiotics-3755903-supplementary.pdf]

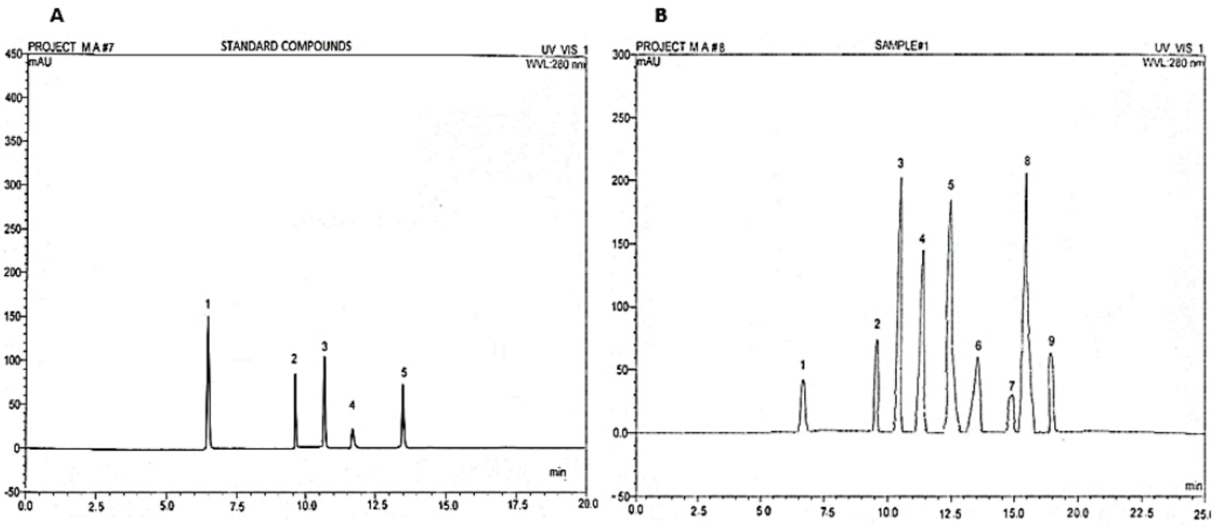

Figure S1. A) Chromatograms of standard phenolic substances at 280 nm wavelengths 1- gallic acid (RT = 6.523); 2- catechin (RT = 9.725); 3-chlorogenic acid (RT = 10.624);4- caffeic acid (RT = 11.731); 5- rutin (RT = 13.524). B) HPLC chromatogram of ethanol extract of guava leaves. Peaks: 1- gallic acid (RT = 6.613); 2-catechin (RT =9.614); 3- chlorogenic acid (RT =10.611), 4- caffeic acid (RT =11.491); 6 rutin (RT = 13.615); (peak 5, 7, 8, 9 are unknown).
